# The Influence of Cardiac Risk Factor Burden on Cardiac Stress Test Outcomes

**DOI:** 10.4021/cr39w

**Published:** 2011-05-20

**Authors:** Jon W. Schrock, Morgan Li, Chidubem Orazulike, Charles L. Emerman

**Affiliations:** a Department of Emergency Medicine, MetroHealth Medical Center, Affiliated with Case Western Reserve University School of Medicine, USA

**Keywords:** Stress test, Coronary artery disease, Cardiac risk factors

## Abstract

**Background:**

Chest pain is the most common admission diagnosis for observation unit patients. These patients often undergo cardiac stress testing to further risk stratify for coronary artery disease (CAD). The decision of whom to stress is currently based on clinical judgment. We sought to determine the influence of cardiac risk factor burden on cardiac stress test outcome for patients tested from an observation unit, inpatient or outpatient setting.

**Methods:**

We performed a retrospective observational cohort study for all patients undergoing stress testing in our institution from June 2006 through July 2007. Cardiac risk factors were collected at the time of stress testing. Risk factors were evaluated in a summative fashion using multivariate regression adjusting for age and known coronary artery disease. The model was tested for goodness of fit and collinearity and the c statistic was calculated using the receiver operating curve.

**Results:**

A total of 4026 subjects were included for analysis of which 22% had known CAD. The rates of positive outcome were 89 (12.0%), 95 (12.6%), and 343 (16.9%) for the OU, outpatients, and hospitalized patients respectively. While the odds of a positive test outcome increased for additional cardiac risk factors, ROC curve analysis indicates that simply adding the number of risk factors does not add significant diagnostic value. Hospitalized patients were more likely to have a positive stress test, OR 1.41 (1.10 - 1.81).

**Conclusions:**

Our study does not support basing the decision to perform a stress test on the number of cardiac risk factors.

## Introduction

Chest pain is a common complaint for patients presenting to the emergency departments in the United States representing over 6 million emergency department visits annually [1, 2]. The initial history, physical examination, and ancillary testing may not be adequate to determine the presence of an acute coronary syndrome in some patients. These patients may need admission to the hospital or more recently to an observation unit for additional monitoring and testing [3, 4].

Patients placed in observation for possible acute coronary syndrome (ACS) often will receive provocative stress testing as part of their workup. The decision to choose who obtains stress testing is less clear. The evidence associated with mandatory stress testing is rated as a level C by some guidelines [3]. Observation unit stress testing has been advocated as cost effective in a population of OU patients based on its ability to reduce hospital admissions [1]. The need for OU stress testing has been questioned based on the low prevalence of disease in young populations and the proposed cost savings with additional testing in a low risk population [5, 6]. Additionally, patients with a very low likelihood of disease who have false positive results may be exposed to more invasive procedures including catheterization and radiation exposure. Further, a prior study has suggested that delayed stress testing is unlikely to be associated with short term adverse results [7].

Cardiac risk factors are used by clinicians to influence the estimation of coronary disease and have been shown to increase the lifetime risk of cardiovascular disease in population-based studies [8]. The utility of these risk factors in ED patients has been questioned with one study finding family history and diabetes useful in men only [9]. A more recent registry analysis found that cardiac risk factor burden was helpful in diagnosing ACS mainly in patients younger than 40 and was not helpful in patients over 65 [10]. It is unclear if cardiac risk factor burden influences the rate of positive cardiac stress testing. It is also unclear if the influence of cardiac risk factor burden changes for patients in observation units, hospitalized patients or outpatients. If there was a positive association of cardiac risk factor burden and stress test outcome it might be useful for physicians who evaluate ACS patients and perhaps help guide diagnostic testing.

We sought to determine the influence of cardiac risk factor burden on cardiac stress test outcome for patients tested from an observation unit, inpatient or outpatient setting.

## Materials and Methods

We performed a retrospective cohort study evaluating all patients undergoing stress testing at our institution over a one-year period from June 30, 2005 through July 1, 2006. After receiving study approval from our institution’s Internal Review Board which included a waiver of informed consent, we created a database of all patients who underwent cardiac stress testing at our institution during the study period. Inclusion criteria included all patients 18 years or older undergoing cardiac stress test evaluation at our institution. Subjects were excluded if they could not undergo the stress test, they were not excluded for early termination of the stress test or for suboptimal stress.

The database contained demographic information, stress test results and listed the presence of the five major cardiac risk factors: smoking, hypertension, hyperlipidemia, diabetes, and family history of coronary artery disease. These risk factors were obtained by the stress testing technician immediately prior to the stress test and were recorded as part of the stress testing results.

Patients were grouped by their location at the time of testing and included OU patients, hospitalized patients, and outpatients. Types of stress testing include exercise treadmill testing, stress echocardiography, and nuclear myocardial perfusion imaging. All stress tests were interpreted by board certified cardiologists. All nuclear imaging results were interpreted by board certified radiologists certified in nuclear imaging. All exercise tests utilized the modified Bruce protocol.

Criteria for positive stress testing included: ST segment down sloping or depression of 1 mm or more during the stress and/or any ventricular tachycardia occurring during the test. Wall motion abnormalities were described as hypokinetic, akinetic or dyskinetic. Nuclear imaging described perfusion defects as fixed or stress induced. Patients undergoing exercise stress testing and failing to meet 10 metabolic equivalents (METs) were considered a suboptimal stress. If signs of ischemia were seen on their stress test they would be considered a positive test. Chemical stress tests examinations used dobutamine, adenosine, or dipyridamole as appropriate.

Final stress test results were provided by a cardiologist and could be normal, abnormal or indeterminate. For the results of this study only those documented as abnormal were considered a positive stress test. Patients with indeterminate or low probability stress test results were classified as normal.

Subjects who had a positive stress test were evaluated to see if cardiac catheterization was performed. Cardiac catheterization was performed at the discretion of the treating cardiologist. If cardiac catheterization was performed, patients with atherosclerotic cardiac lesions of 50% or greater were considered to have obstructive coronary artery disease (CAD). Rates of coronary artery bypass grafting (CABG) and percutaneous transluminal coronary angioplasty (PTCA) were recorded.

Results were reported as frequencies and medians with interquartile ranges (IQR) where appropriate. Logistic regression was performed for the sum of cardiac risk factors from one to five based on location and as a group, controlling for age > 60 and known prior coronary artery disease at the time of stress testing. Patients were defined as having prior coronary artery disease if they had a prior myocardial infarction, coronary artery bypass graft procedure (CABG), or prior coronary artery disease found on prior cardiac catheterization. Logistic regression was used to compare the odds of a positive stress test based on patient location using outpatients, the largest group, as a reference. Odds ratios are reported with 95% confidence intervals. The Hosmer-Lemeshow test was used to test the model for goodness of fit with P < 0.5 suggesting poor fit. Collinearity was tested using regression diagnostics to evaluate the study variables with a condition index of 30 or greater suggesting severe collinearity. Data were incorporated into a database using Excel 2003 (Microsoft Inc., Redmond WA). Statistical analysis was performed using STATA version 11.1 (College Station, TX).

## Results

**Figure 1:**
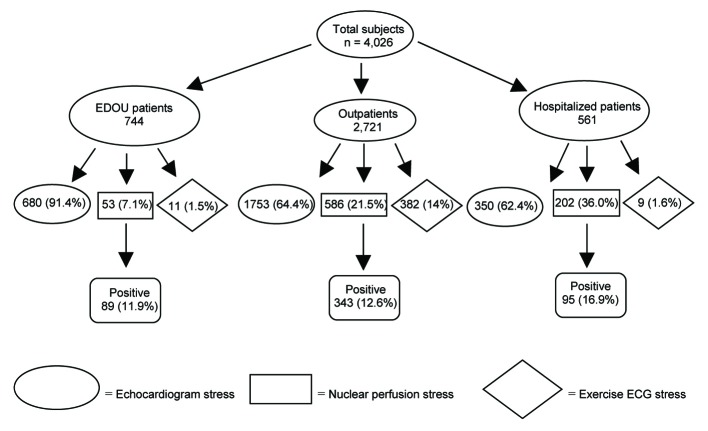
Distribution of stress tests based on patient location and type of test.

A total of 4,040 patients were evaluated of which 14 did not undergo stress testing and were excluded from the study allowing 4,026 subjects for analysis. The median ages based on patient location were 49 yrs (IRQ 45 - 57 yrs), 55 yrs (IQR 47 - 64), and 56 yrs (47 - 67 yrs) for OU, outpatient, and hospitalized patients respectively and 1,730 (43%) were male. The frequency of subjects with prior known coronary artery disease was 866 (22.0%). The types and distribution of stress tests can be seen in Figure 1. The number of indeterminate stress test results for the OU, outpatients and hospitalized patients were 50 (6.7%), 232 (8.5%), and 47 (8.4%) respectively. A total of 527 (13%) stress tests were abnormal. The number of positive stress tests based on location was 89 (12.0%), 95 (12.6%), and 343 (16.9%) for the OU, outpatients, and hospitalized patients respectively. The odds of a positive stress test based on patient location using the outpatient group as a reference was 0.94 (0.73 - 1.21) and 1.41 (1.10 - 1.81) for the OU and inpatients respectively.

The multivariate logistic regression showed increased odds of a positive stress test for each additional risk factor for the group overall (Table 1). These results showed a small incremental increase of the odds ratios when the regression was adjusted to control for patients aged 60 and older (Table 2). A steady increase in odds ratios was seen in the OU group with the other groups showing little change between 2 and 3 risk factors. The regression was tested using the Hosmer-Lemeshow test which showed excellent fit with a P = 0.90 for the model. The condition index was 4.9 which suggested little collinearity among the variables tested.

**Table 1:** Logistic Regression for Cardiac Risk Factors Based on Patient Location and for the Group

Number of risk factors	Overall N = 4,046 (95% CI)	OU N = 45 (95% CI)	Inpatient N = 561 (95% CI)	Outpatient N = 2,740 (95% CI)
1	1.6 (1.1 - 2.2)	1.6 (0.8 - 3.3)	0.6 (0.2 - 1.7)	1.8 (1.2 - 2.7)
2	2.3 (1.7 - 3.2)	2.2 (1.1 - 4.5)	1.7 (0.8 - 3.8)	2.4 (1.6 - 3.6)
3	2.5 (1.8 - 3.4)	3.8 (1.9 - 7.5)	1.6 (0.7 - 3.3)	2.4 (1.6 - 3.6)
4	4.0 (2.8 - 5.7)	5.5 (2.3 - 12.8)	3.3 (1.3 - 8.0)	3.5 (2.2 - 5.6)
5	5.2 (2.3 - 11.7)	*	7.4 (1.6 - 34.4)	5.4 (1.8 - 15.9)

* Groups 4 and 5 combined. Odds ratio for positive stress test based on number of cardiac risk factors

**Table 2:** Odds of a Positive Stress Test Based on Cumulative Risk Factors and Adjusted for Age and Prior CAD

Number of risk factors	Odds ratio (95% CI)
1	1.5 (1.1 - 2.1)
2	2.0 (1.5 - 2.8)
3	2.1 (1.5 - 2.9)
4	3.1 (2.0 - 4.5)
5	3.7 (1.2 - 8.7)
Age > 60	1.5 (1.2 - 1.8)
Prior CAD	1.4 (1.1 - 1.7)

**Figure 2:**
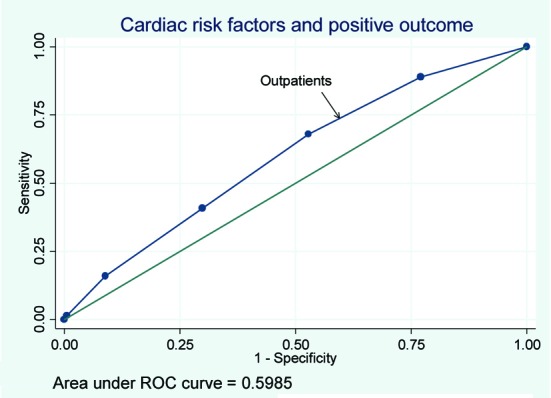
The receiver operating curve for outpatients.

**Figure 3:**
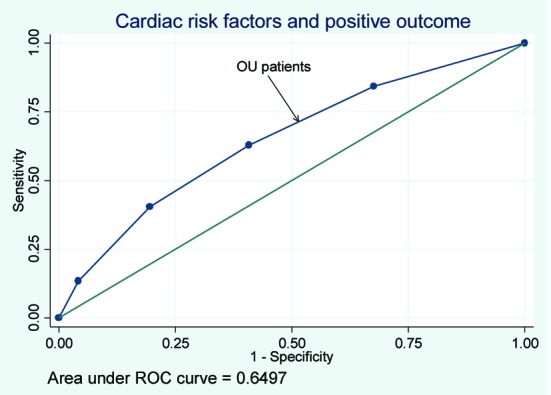
The receiver operating curve for OU patients.

**Figure 4:**
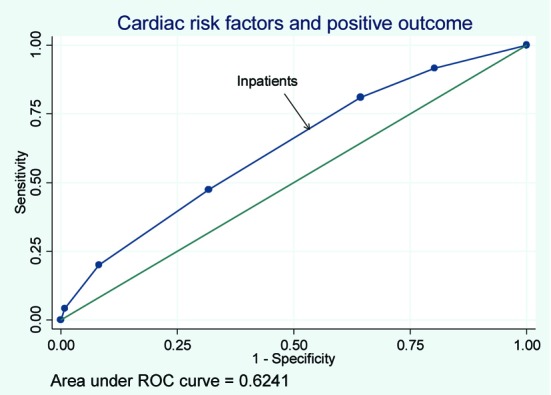
The receiver operating curve for inpatients.

Of the patients with positive stress tests, rates of cardiac catheterization for the OU, outpatients, and hospitalized patients were 37%, 33%, and 41% respectively. Rates of interventions for the three groups can be seen in Table 3. The rates of PTCA and CABG were similar among the three groups. The rates of PTCA and CABG were lowest in the OU group when compared with the inpatient and outpatient groups. The receiver operating curve for risk factor burden and a positive stress test for patients based on location were derived resulting in a c statistic of 0.59, 0.65, and 0.62 for outpatients, OU patients and inpatients respectively (Fig. 2, 3, 4).

**Table 3:** Outcomes of Patients Taken for Cardiac Catheterization Based on Patient Location

Procedure	EDOU N = 33 (%)	Outpatient N = 113 (%)	Inpatient N = 39 (%)
PTCA	1 (3)	19 (17)	2 (5)
CABG	5 (15)	21 (19)	8 (21)

## Discussion

Chest pain is one of the most common complaints for patients presenting to emergency departments [11, 12]. Many of these patients are admitted either the hospital or to OUs for serial testing and observation. Part of their evaluation includes risk stratification and if deemed appropriate, cardiac stress testing. Having useful tools to adjust risk of CAD for these patients would be helpful in choosing additional testing.

We found that as cardiac risk factor burden increased so did the odd of obtaining a positive stress test. This was true even after adjusting for age and did not differ significantly if the patient being tested was an outpatient or hospitalized patient. These results are in agreement with other studies that have found that the rates of ACS increase with increased number of cardiac risk factors [8, 10]. It would make clinical sense that increased number of risk factors would increase the odds of a positive stress test. On the other hand, the area under the ROC curve shows that just adding the number of risk factors does not substantially improve the diagnostic value.

The area under the ROC curve, the c statistic, was 0.63 which would be considered poor. While additional cardiac risk factors will increase the odds ratio of subsequently having a positive stress test, the ROC test results suggest that using this model would not be helpful as a clinical decision tool. These results suggest that while additional risk factors increase the odds ratios for a positive stress test they perform poorly as a decision making tool.

The lack of influence of the odds ratios on the c statistic, may seem counter intuitive. However, one property of the c statistic is that for a risk marker evaluated in isolation, large odds ratios are needed to create clinically meaningful changes [13, 14]. This does not mean that combinations of independent odds ratios or relative risks with small are not useful in creating risk scores. The Framingham risk score, a tool to assess the 10-year risk of cardiovascular events in patients, was derived from variables with relative risks less than 3 but still performed well with a c statistic of 0.74 in men and 0.77 in women [15]. It would be interesting to evaluate the results of risk factor burden in conjunction with other testing that would be predictive of positive stress tests. Some inflammatory markers, such as high sensitivity C reactive protein have been evaluated in this setting with mixed results [16, 17]. Other markers such as N-terminal prohormone brain natriuretic peptide (NT pro-BNP) have been suggested to hold promise as a potential marker for increased risk of an abnormal stress test but again preliminary results are conflicting [18, 19].

We did not use the Framingham risk score in our analysis because many patients did not have serum lipid testing at the time of testing which is a required component of the score. Using the score would have led to a large number of subjects with missing data.

The amount of emphasis which should be placed on clinical risk factors has fluctuated over the years. In the 1990s several reports were published estimating that a large minority to half of all patients diagnosed with coronary heart disease did not have classic risk factors [20-22]. This led to an increase in research evaluating other potential risk factors such as inflammatory biomarkers. More recent research suggests that the role of conventional cardiac risk factors was higher than had been suggested [23, 24].

While it seems clear that cardiac risk factors have an association with CHD it is not clear how to use that information. Our study suggests significant trends exist for an increased risk of an abnormal stress test with increasing number of cardiac risk factors. The association, while present, is not significant enough to guide therapeutic decisions alone.

This study has several limitations. It was conducted at a single institution and may be limited by selection bias of presenting patients. Rates of diagnostic testing have been shown to vary based on geography which may alter our results [11, 25]. Our outcome of positive stress test while useful may not always equate to an outcome of an adverse cardiac event such as cardiovascular death or myocardial infarction. We choose to classify stress tests resulting in low probability or indeterminate as not positive stress tests to avoid dilution of the effects of cardiac risk factors on the presence of coronary disease.

Stress tests may be ordered for other reasons besides acute evaluation of ACS including pre operative testing, cardiac evaluation after CABG, and evaluation of other diseases such as heart failure. Some patients may have testing ordered for alternative reasons which may have potentially affected rates of intervention.

We did not evaluate other forms of testing such as cardiac computed tomography angiography (cardiac CTA) or patients taken directly to cardiac angiography as this was not the objective of the study. We relied on patient revelation to determine the number and type of risk factors for each patient. Some patients may be unaware of a risk factor such as family history of myocardial infarction or hyperlipidemia. We treated all cardiac risk factors easily and did not weight them in our analysis as clinicians using them would likely treat them all equally.

In conclusion we found that an increasing number of classic cardiac risk factors were associated with an abnormal cardiac stress test for all patients. The likelihood of a positive stress test increases as the number of risk factors increases. Despite this increased likelihood, the diagnostic value of adding the number of risk factors is not high enough to use it as a decision tool as measured by the c statistic. There were not significant differences between the OU and other groups in the percent of positive stress tests or the association between risk factors and positive tests. This suggests that clinical decision making is still important in determining who should get a stress test rather than a mechanical system of adding up risk factors. We cannot recommend using the number of cardiac risk factors in guiding decision making in predicting which patient may have a positive cardiac stress test. Future studies utilizing cardiac risk factors with other risk stratification tools such as biochemical markers may hold more promise guiding clinical decision making.
